# Using intergenerational photovoice to understand family strengths among Native American children and their caregivers

**DOI:** 10.1002/jcop.22860

**Published:** 2022-04-11

**Authors:** Katie M. Edwards, Ramona Herrington, Marcey Edwards, Victoria Banyard, Natira Mullet, Skyler Hopfauf, Briana Simon, Emily A. Waterman

**Affiliations:** ^1^ Nebraska Center for Research University of Nebraska‐Lincoln Lincoln Nebraska USA; ^2^ School of Social Work Rutgers University Newark New Jersey USA; ^3^ Psychology Bennington College Bennington Vermont USA

**Keywords:** adverse childhood experiences, American Indian, childhood abuse, family, intergenerational, Native American, photovoice, strengths

## Abstract

The purpose of the current study was to examine Native American children and caregivers' perspectives of family and cultural strengths using photovoice and to identify lessons learned from the first‐ever implementation of intergenerational photovoice with Native Americans. Participants were Native American, low‐income caregivers (*n* = 6) and their children (*n* = 12) between the ages of 10 and 15 who participated in six photovoice sessions. The themes that emerged from photos and group discussion included myriad challenges faced by Native American families including exposure to community violence, substance abuse, and criminal offending and incarceration. Themes also emerged that highlighted the strengths of Native families that were used to overcome identified challenges, including religion/spirituality, engagement in traditional cultural practices (e.g., prayer, song, dance), healthy activities (e.g., running, meditation). These data provided foundational information that is currently being used, along with other data, to develop a culturally grounded, strengths‐focused, family‐based program (*Tiwahe Wicagwicayapi* [Strengthening/Growing Families in Lakota]) to prevent adverse childhood experiences. We also discuss the challenges of intergenerational photovoice and lessons learned to inform future intergenerational photovoice projects.

## 1 INTRODUCTION

Research documents the myriad health disparities experienced by Native Americans (Adakai et al., 2018; Dickerson et al., 2019; Kaufman et al., 2020). Adverse childhood experiences (ACEs) are one such health disparity (Warne et al., 2017). ACEs are traumatic or upsetting experiences that happen before the age of 18 and include things such as child maltreatment, exposure to parental mental illness, and exposure to community violence (Centers for Disease Control and Prevention Prevention, 2019). For example, 83% of Native American adults reported one or more ACE, compared to 50% of nonnative adults. ACEs are associated with myriad short‐ and long‐term negative health outcomes and cost $748 billion in the United States in annual healthcare costs (Bellis et al., 2019; Centers for Disease Control and Prevention Prevention., 2019). Given the high rates and negative outcomes associated with ACEs, prevention of ACEs is needed. To date, however, we know little about how to prevent ACEs, especially among Native Americans.

The CDC suggests that one likely effective strategy to prevent ACE, especially violence‐related ACEs, is to ensure strong starts for children and enhance family strengths such as family bonding and communication as well as parental monitoring (Centers for Disease Control and Prevention Prevention., 2019). The CDC's suggestion aligns with calls to move away from “damaged‐centered” research on Native Americans (Tuck, 2009), and instead focuses on the individual, family, and cultural strengths of Native Americans (Craig, 2019; Hamby et al., 2020; Yuan et al., 2015). An understanding of family strengths from the perspectives of Native American children and caregivers will likely provide important information that can be leveraged to create strengths‐focused, culturally grounded, family‐based prevention initiatives.

It is important that research with Native Americans be community‐led and participatory (Yuan et al., 2014). It is also important that research with Native Americans use Indigenous ways of knowing, which may include arts‐based methods such as photovoice (https://pubmed.ncbi.nlm.nih.gov/35249402/). In the current study, a group of Native American community members in conjunction with university‐based researchers selected photovoice as the preferred method of documenting family strengths. Photovoice is a participatory action research method that engages minoritized groups in the exploration, identification, and critical analysis of a problem that they face (Strack et al., 2004; Wang & Burris, 1997). Participants use photographs and group discussions as a tool to analyze the causes of the problem, reflect on personal meanings of the problem, imagine solutions to the problem, and share their solutions with an audience of their choice, most commonly the broader community (Lightfoot et al., 2019; Woods‐Jaeger et al., 2013). At the conclusion of photovoice, participants share their photographs and their meanings as part of a community exhibit to not only raise awareness about the problem but to make specific calls to action.

In the current study, at the request of Native (Lakota) community members, participants engaged in intergenerational photovoice that included both children (ages 10–15) and their caregivers. Previous photovoice projects have overwhelming included members of a single generation. As described by a Lakota elder, “A cornerstone of Lakota culture can be summed up in the words of family and kinship. Family is the backbone, the foundation of our culture.” In Lakota culture, the family (*tiwahe*) and extended family (*tiyospaye*) play a critical role in supporting the healthy development of children (*wakanjeja*). In Lakota culture, children are considered sacred and a gift from the Creator. Children learn about the Lakota virtues from their *tiwahe* and *tiyposaye*, which include: humility (*wahwala or wounsiciye*), perseverance (*wowacintanka*), respect (*waohola*), honor (*wayuonihan*), love (*canteognake*), bravery (*woohitike*), fortitude (*cantewasake*), generosity (*wacantognaka*), and wisdom (*woksape*). These virtues are foundations of Lakota culture and contribute to individual, family, and community strengths, and are believed to protect against adversities, including violence (Edwards et al., 2020; Freeman et al., 2016). Thus, a photovoice project that includes multiple generations rather than children or adults separately seemed most consistent with community and cultural values.

Few studies have used intergenerational photovoice and only one to our knowledge with Native Americans. Previous intergenerational photovoice topics have focused on health promotion (Gabel et al., 2016; Garcia et al., 2013), crime prevention (Ohmer & Owens, 2013), and environmental justice and land sovereignty (Carroll et al., 2018). These projects illustrated enhanced understanding that comes from including voices across generations. For example, research conducted by Gabel et al. documented that elder‐youth interactions in photovoice helped to facilitate the creation of interventions focused on enhancing and supporting intergenerational relationships, thus supporting cultural continuity and overall well‐being.

Previous photovoice projects (that were not intergenerational) with Native Americans have focused on water and health (Mitchell, 2018), indigenizing libraries (Beatty et al., 2020), and sexual violence (Banyard et al., 2020). In the Banyard et al. (2020) photovoice project on sexual violence, the sample was largely Native, and a key theme was that despite newfound freedoms associated with adolescence, youth still needed support from others, including their families to have healthy relationships. Other photovoice projects with adults have focused on experiences of violent victimization and healing and recovery processes, although none of this study has been intergenerational and/or focused specifically on Native Americans (Christensen, 2019). These published intergenerational photovoice projects also did not include detailed information on implementation challenges and reflections on lessons learned which may be helpful to individuals wishing to implement future intergenerational photovoice projects with Native American families.

The purpose of the current study was to examine via photovoice Native American children and caregivers' perspectives of family challenges and how they use individual, family, and cultural strengths to overcome those challenges. These data were collected as part of a larger project to develop a strengths‐based, culturally grounded, family‐based program (*Tiwahe Wicagwicayapi* [Strengthening/Growing Families in Lakota]) to prevent ACEs among Native Americans. Photovoice was one of several methods used to guide the development of the program. An additional aim was to reflect on lessons learned to conducting the first‐ever intergenerational photovoice study with Native American caregivers and their children.

With regard to positionality, the facilitators of photovoice, who are co‐authors on this paper, included a Lakota grandmother and a Lakota mother. The facilitators were long‐time residents in the small city where this study took place and enrolled members of nearby tribal nations. The researchers spent time immersed in the community and the research team had worked together with community members for several years before this project was initiated. What is more, we collectively represent a team comprised of White, non‐Hispanic, Hispanic, and Native American individuals, queer and heterosexual cisgender individuals, ranging from young people to elders, that include graduate students, community‐based research staff, community activists, prevention specialists, and individuals working in higher education including faculty. Together, we are committed to developing and evaluating ACE prevention and response efforts as well as efforts to prevent substance abuse, homelessness, food insecurity, and school drop‐out among Native Americans. Further, we believe that interpersonal violence, including ACEs, and other public health and safety issues must be understood within a sociopolitical and historical lens, that community based participatory action research (CBPAR) is the most effective way to prevent and respond to interpersonal violence, and that researchers' power and privilege, including white privilege, must be acknowledged and dismantled through reflexivity and a commitment to antiracism work.

## 2 METHODS

### 2.1 Participants

Participants were (*n* = 6) caregivers (*M* age = 49.3; SD = 8.5; range = 37–60) and their children (*n* = 12) between the ages of 10 and 15 (*M* age = 12.6; SD = 1.7). Participants were all Native Americans residing in a small city in the Northern Plains region of the United States in close proximity to several large tribal nations. Caregivers were 100% women; youth were 50% boys and 50% girls. All caregivers reported that their yearly income was less than $40,000 per year (two‐thirds reported less than $10,000 per year).

### 2.2 Procedures

Families were recruited via flyers posted throughout the community, word of mouth, and social media. Participants met weekly for 6 weeks; each session was 2 h. Following each session, participants were provided with a $15 gift card. Participants were also served dinner at each session and kept their digital cameras following the end of the photovoice.

Following procedures of previous photovoice projects (Gabel et al., 2016; Garcia et al., 2013; Sidibe et al., 2018), the first session focused on (1) rapport building, (2) providing an overview of photovoice (including an example from a previous photovoice project in the community that focused on sexual violence and conducted by this team; https://onlinelibrary.wiley.com/doi/abs/10.1002/jcop.22495) and the larger project that focused on ACEs prevention, (3) agreements and ground rules, (4) technical training on how to use cameras, and (5) selecting the first photovoice homework assignment. Children and caregivers were together during the first session.

During sessions two through five, children first met with one another while caregivers first met with one another (45 min) and then all participants came back for a group discussion during the last part of the session (45 min); the remaining 30 min of the 2‐h session including check‐in and check‐outs, dinner, and time for small talk.

During sessions two through five, in both the smaller groups and larger group, participants shared and discussed photos using the “SHOWED” method (Lightfoot et al., 2019). The SHOWED method includes sharing what participants saw in each photo, how they felt it related to their and others' lives, why things are this way, and how they can educate others and create change about the issues they see represented in the photos (see Table 1 for more details). Facilitators asked probing questions (e.g., “tell me more about that” and “what do others think” to elicit more details). There was sufficient time in all the sessions to go through all the photos shared by participants (not every participant brought a photo each week). In other words, the children first went through the photos that they took together using the SHOWED method (while the caregiver went through the photos, they took together using the SHOWED method), and then the children and caregivers came back together and went through the SHOWED method together for all the photos. At the end of sessions one through four, participants decided together on the theme for the following week (see Table 2).

**Table 1:** SHOWED method overview

Questions asked for each photo discussed
What do we See in the photo?
What is Happening in the photo?
How does this photo relate to Our lives?
Why do these issues exist?
How can we become Empowered by our new understanding? Empowered means strong and proud!
What can we Do to address these issues?

**Table 2:** Weekly photovoice themes (as determined by participants)

Session	Theme
1	N/A
2	How families show love
3	How families de‐stress
4	How to keep families safe
5	How to bring peace and happiness to families
6	N/A

The final and sixth sessions focused on planning for the community exhibit and reviewing themes and photos for this paper. The participants who missed session six were invited to meet with the facilitators to help provide input on the photos and quotes selected for the exhibit and this paper. During these meetings, participants reflected on what they saw as the impact of the photovoice project on themselves and their community.

Session attendance ranged from 6% of participants to 100% of participants. There was an average of 3.2 photos discussed during sessions two through five, and all 16 photos were discussed at session 6. All sessions were audio‐recorded with guardian consent and youth assent and transcribed verbatim for analysis (with identifying information removed). All research procedures were approved by the University of Nebraska‐Lincoln Institutional Review Board (IRB).

### 2.3 Data analysis

We used an iterative process to analyze data. First, facilitators started and ended each session by having participants summarize key themes from current and previous sessions. These themes were discussed with the research team on a regular basis while photovoice was underway following analysis procedures described by Lightfoot and colleagues. The first and fifth authors also observed photovoice sessions via Zoom or in person, and the first author reviewed transcripts during and after photovoice. The first author then worked with the facilitators (second and third authors) to reread all the transcripts and identified themes across sessions that emerged within the child, caregiver, and family sessions. These themes were discussed with participants in session six and individual follow‐ups, a form of member checking. The decision was made to have the research and facilitator teams conduct data analyses rather than participants (see Foster‐Fishman (Foster‐Fishman et al., 2010) for examples of more participant‐centered data analysis) given expressed time constraints by participants.

### 2.4 Photovoice results

Themes centered around challenges and strengths. Whereas only caregivers identified challenges, both children and caregivers discussed family strengths as delineated below.

### 2.5 Challenges

Whereas most of the sessions focused on family strengths, it was important to caregivers to have a space to discuss family challenges. This also provided them a foundation to think about the ways in which they used family strengths to overcome challenges. Although caregivers did not take photographs of family challenges (as their photographs focused on family strengths), caregivers discussed incarceration, substance abuse, and exposure to community violence. For example, one caregiver said:

*One of the things that I think about and worry about for the future for [my children is that they could] end up in jail, end up in prison. I think about that. I think about my nephews and my nieces, some of them are in prison, so I always think what I can do. Thank God my kids aren't [in prison].*

Several grandmothers reflected on taking care of their grandchildren due to parental absence related to substance abuse and/or incarceration. One woman said:

*She chose the drug over her kids. I raised her kids. I still have them. I told them that's your mom, so they do call her mom. She's out now, she says she's sober, but I know [she is] still using.*

Caregivers also talked about exposure to violence, especially community violence. One caregiver said:

*When I go outside of town, I see all the gang stuff. You see them in their colors and going around. I think that [the community] needs to address that, because first of all, I look at it like this, that's not our culture. I think they need to start saying that.*

Another caregiver stated: “Somebody was getting beat up right outside my house. There was all kinds of stuff that would happen. It happened all the time.”

### 2.6 Strengths

#### 2.6.1 Strengths from past adversity

Among caregivers, an important theme that emerged connected their discussion of challenges with strengths. Many described the desire to be better people (make better decisions, be role models) for their children and grandchildren who served as a source of strength and resilience for them. For example, one caregiver said:

*I think having a drug and alcohol‐free home is really important to families that want to raise their kids the right way. I want to raise my grandkids the right way. I made mistakes being a parent to my kids. I'm trying to change for my grandkids. I've been sober over 20 years now and it was hard, but it's worth it, though.*

Another grandmother said: *I think my grandkids empower me to be strong. I used to drink a long time ago, [but] they empower me to straighten up.*

**Figure 1:**
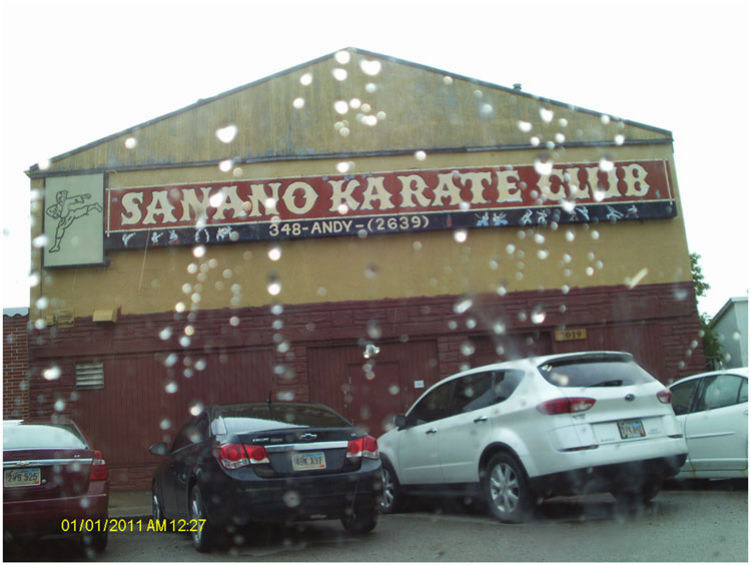
I believe families should all know self‐defense. In these times it is important. It keeps the family safe. Martial arts teach discipline. Nobody should ever walk alone. I worry for the children and want to do everything I can to teach them to be safe

Another theme that emerged among caregivers that focused on the importance of keeping children safe. This included things such as “mak[ing] sure we lock our doors before we leave just in case someone tries to steal [our] car.” Caregivers also talked about the importance of knowing where their children were. For example, one caregiver stated: “knowing where they're at having them safe. And they're teenage girls and it's just I need to know where they're at.” Similarly, another caregiver said: “But just for me to feel safe, I like when we're all keeping track of each other.” For many, their home was their safe space, despite concerns that there were high rates of community violence. A final theme that emerged was teaching children to protect themselves as depicted in Figure 1. In addition to the quote displayed in Figure 1, another caregiver said:

*Teaching our children how to defend themselves will definitely keep them save. Abductions and that thing. And it also teaches them self‐discipline, so they would be more likely be more respectful to the elders and listen and learn more easily because of the self‐discipline that they've got.*

#### 2.6.2 Family strengths

Themes also emerged that highlighted the strengths of Native families that were used to overcome challenges which included religion/spirituality and engagement in traditional cultural practices (e.g., prayer, song, dance). Prayer was an especially prevalent theme, especially among caregivers. Prayer was seen as “keeping families strong.” As another participant noted:

*Dancing is prayer. We talked about when they do the jingle dress dances, that's healing. They do that for healing. Making the best choice. Grandkids have empowered me to be strong.*

**Figure 2:**
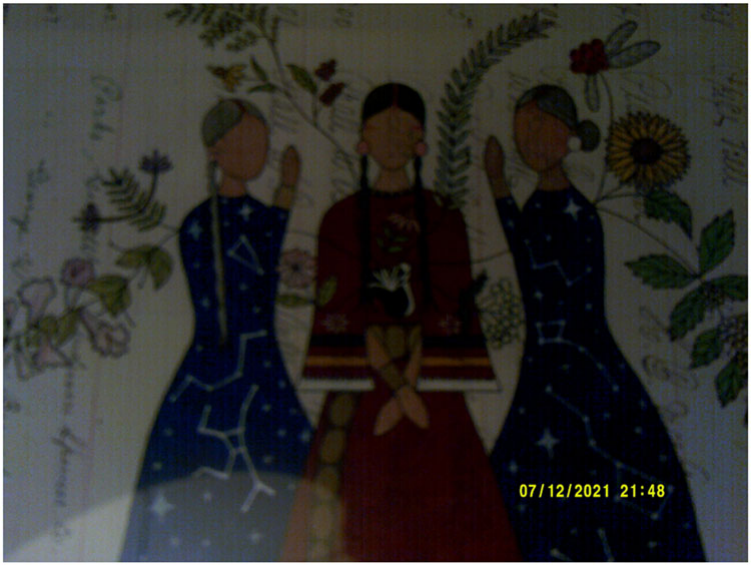
Safety is the presence of connections. Plant medicines around. What we need is healing. We are connected. Lakota relatives being strong together—our spirituality, prayers, and songs make it feel safe. An intergenerational home with grandparents, parents, and grandchildren giving and sharing

**Figure 3:**
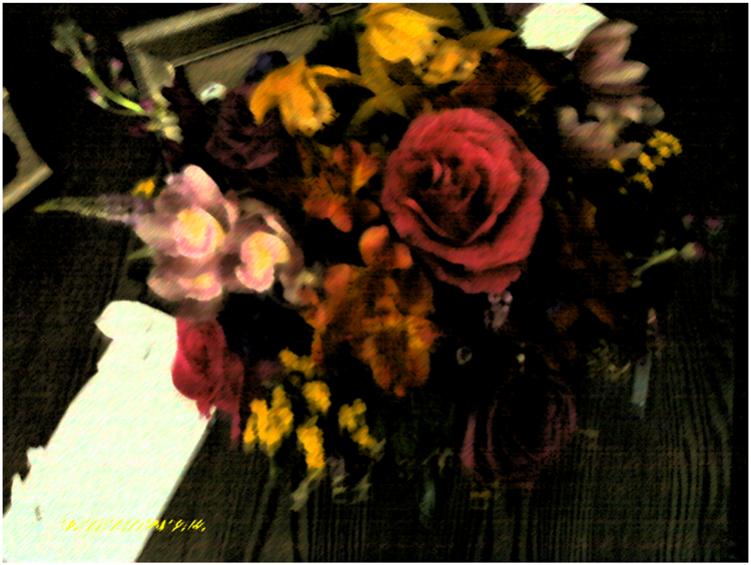
We are all different in ways [yet] we all shine as a family. [We have] beauty of a family. Thorns could be strong, [and] every bad day only lasts 24 h. Do not feed into negativity. As a family, we need to pray together more

Another child stated, “when you pray it helps” and a caregiver said, “spirituality keeps us together.” Figures 2 and 3 further demonstrate the role of culture in helping families to feel strong, specifically the role of prayer, dance, and song as well as the importance of families across generations being physically and emotionally close to one another. As another caregiver stated:

*The roots can be like the center of your family and the stronger you get, the more better the roots grow. As the season change, so your family might be going through problems, the leaves fall off. But they're going to… seasons come and go and no matter what happens‐like the trouble you're having at home, at school‐the leaves will come back and it will turn green again and it will flourish and prosper.*

Participants also reflected on healthy activities (e.g., running, meditation) that help them to relieve stress, which ultimately supports family strengths. Children stated “[*being outside] is a quiet place to calm down*” and another child expanded on that by stating “*because you're out in the air*.” A caregiver said: *Running is meditation, so that's like prayer*. Another caregiver stated:

*I was out in the hills, and so I took some pictures over there. For me, just taking a walk, away from all the chaos of this city, really helps me, and I do like a walking meditation where I just focus on what's around me, you know? And it helps. It helps, because my mind will always be working, so it kind of gives my mind peace.*

**Figure 4:**
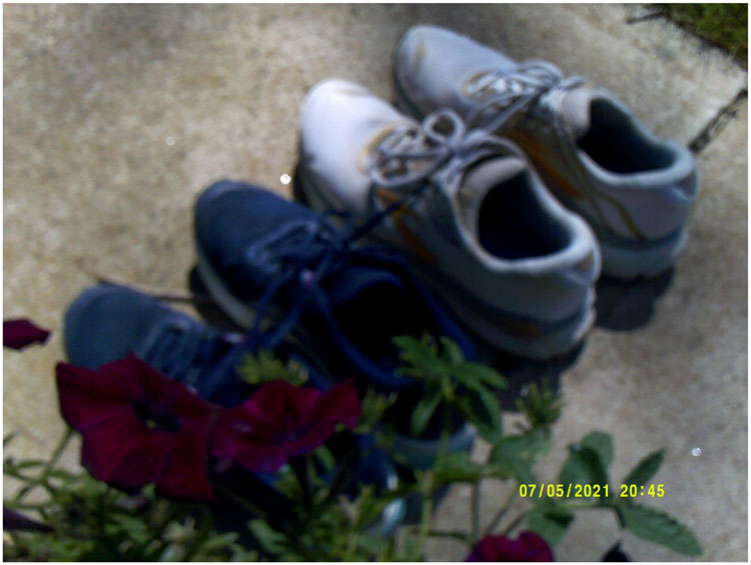
I feel at peace and ready to run when I see the flowers, quality time with my son, it is an honor to run with my son, running is healing. It makes my heart less heavy; it is uplifting and empowering

As reflected in Figure 4, healthy activities were especially powerful when they included one's child.

Beyond exercise and being outside families identified things that they liked to do with one another that promoted family strengths such as “gather[ing] in the living room to enjoy family time or just to sit and talk, maybe do puzzles” and “We use music to soothe us.” Children also talked about doing good things for other people such as “sometimes we go and clean up the neighborhood” and “it helps mother nature.”

Finally, participants reflected on the importance of eating meals together. One participant stated:

*I think one of the things that was really a big difference for us was when we moved to our house on the south side, we didn't have a big dining area. We used to always eat all together. That really made a big change in my family. It was the kids, the boys even said that. “Remember when we used to eat together?” And I said we still eat together. And they said, “Yeah, but we don't sit down and look at each other.” And I think that makes a big difference.*

### 2.7 Community exhibit

The community exhibit took place in October 2021, approximately 2 months following the conclusion of photovoice (in August 2021), and coincided with a community feed that marked the launch of recruitment for the program that was created using data from the photovoice sessions as well as other data sources (e.g., focus groups with children and caregivers [outlined in Section 3 of this paper]). The community feed was open to the entire community and took place at a local Native American‐run church in a part of town where mostly Native American families reside. The photos and corresponding quotes were on display at the entrance of the church for families to view as they came to the feed and a local news station was present to do a story on the feed, that included the photovoice project. The photos have remained on display in the church during sessions of the *Tiwahe Wicagwicayapi* program and other community events (e.g., Holiday feed, December 2021). The photos have been shared in other community outlets (e.g., Advisory Board meetings, and community events). In all, it is estimated that over 300 community members have seen the photos to date.

### 2.8 Lessons learned

Many lessons were learned as part of this project, which was the first intergenerational photovoice with Native American children and their caregivers. A number of participants noted that they enjoyed being in the project and felt like it afforded them the opportunity to spend quality time with one another and enhanced their emotional bonds. In fact, some adult participants reported anecdotally to the photovoice facilitators and researchers that they thought photovoice felt like an intervention to help with family bonding and cohesion, which subsequently helped in other areas of their lives (e.g., improved mood). Although other researchers have speculated that photovoice may, indeed, be an intervention (Werremeyer et al., 2020), this is merely speculative in the current study but warrants further investigation in the future about the utility of photovoice as a potential source of support for Native American caregivers and their families. This may be especially true for urban Native Americans (such as those in the current study) where there are often few opportunities for ongoing engagement in Lakota cultural activities.

Despite the information gleaned in this study, some challenges also arose, especially regarding participant engagement. There is a growing body of literature documenting the challenges of engaging Native Americans in research (Baldwin, 1998; Yuan et al., 2014, 2015) including violence‐focused research (https://pubmed.ncbi.nlm.nih.gov/35249402/). Many of the challenges (e.g., trust, exploitation) can be addressed using Indigenous‐led, CBPAR approaches. However, that is not always the case.

Indeed, the current study used CBPAR, but we still faced a number of challenges with participant engagement. First, the project took place during the COVID‐19 pandemic. Despite the vaccination being widely available, COVID still impacted either directly or indirectly a number of the families involved, which impacted their ability to attend sessions. Online attendance was not a viable option for many families, who did not have easy access to the Internet and computers. What is more, participating families also could not make sessions at times due to deaths in the family and transportation challenges. Indeed, many participants expressed difficulties with finding consistent transportation to sessions. Some participants arrived late to sessions due to having to wait on transportation after work. Further, some participants expressed not having money to pay for gas to get to the photovoice sessions, and some participants found it difficult to get their work schedules to align with the time of the sessions. Some participants shared that they had to move shifts or ask for time off from work which was not an option. For the children, some school activities interfered with photovoice sessions and prevented them from attending. Finally, some families also had vacations planned which overlapped with the study.

These challenges suggest that future photovoice sessions with Native American families need to be flexible with scheduling, secure funding to assist with transportation to sessions and offer alternative modes of participation. One option would be providing families with tablets and data/Wi‐Fi, increased incentives to cover for time missed at work, and grant funding that would allow for the use of a vehicle to transport participants to sessions (for those who wished to attend in person). All of these are costly things to implement yet would likely enhance the ability for Native American families to fully participate in photovoice. These may be especially relevant for urban Native Americans who transient, which is frequently back and forth between an urban area and nearby Indian reservations, which was the case for many of the participants in this study. It would be important, however,

Another lesson learned from this project is that intergenerational photovoice may benefit from being longer than six sessions, especially since we found that it took children several sessions before they felt comfortable enough to share. Indeed, during the first few sessions, many of the children were quiet and hesitant to participate. However, after they became more comfortable with the facilitator and the other participants, they begin to share without hesitation. Having additional sessions would also provide families who had to miss sessions due to personal reasons (e.g., family illness) with additional opportunities to participate.

A final point of reflection relates to staff roles and project inclusion criteria. First, the project required moderate to high levels of technology skills that were also time consuming. Thus, rather than have the photovoice facilitator take on this burden (while trying to do their other job responsibilities), we had another individual present at all photovoice sessions to assist with the set‐up of the projector and laptop, transfer of photos from the individuals' cameras to the computer, and so forth. Although we tried to hire a Native individual to fulfill this duty, we were unable to do so, and there was some concern among our Native facilitators that it might have made Native participants uncomfortable, or seemed odd, that a White, non‐Hispanic man was present at the beginning of sessions although he left after the technology pieces were set up. Another important point worth noting is that some participants would occasionally want to bring a member of their extended family (*tiyospaye*) either due to necessity (e.g., they were babysitting their niece that day) or because they felt it would be meaningful for that individual to participate (e.g., their sister was struggling and felt like she could benefit from coming to photovoice). However, due to the need for legal guardian permission for children to participation, the importance for both children and their caregiver to be present, and the desire for there to be consistency in participants across sessions, we were not able to accommodate all requests for new attendees. Taken together, future photovoice projects should consider the technology needs of the project (and hire staff from within the community, if possible, to help address those needs) and explore ethical and meaningful ways to engage participants who are part of larger *tiyospayes* in more time‐limited ways.

## 3 DISCUSSION

The purpose of the current study was to examine via photovoice Native American children and caregivers' perspectives of family challenges and how they use individual, family, and cultural strengths to overcome those challenges. Similar to other research with Native Americans (Warne et al., 2017), participants (especially caregivers) discussed the many adversities that they experience, namely violence, substance abuse, incarceration, and parental absence, all of which are ACEs (Centers for Disease Control and Prevention Prevention., 2019). It is important to acknowledge that these adversities did not exist in traditional Lakota society and were introduced only after colonization (Marshall, 2002). The devastating impact of colonization and multiple historical traumas (e.g., genocide, boarding schools) perpetrate these adversities and underscore the critical need for structural and systemic changes that reduce racism and poverty, structural determinants of these adversities (Paradies et al., 2015; Trent et al., 2019; Warne & Lajimodiere, 2015).

At the same time, efforts are needed to enhance family strengths which may help to prevent some forms of ACEs and buffer against the deleterious effects of ACEs exposure. In the current study, connection to culture was identified as an important family strength among participants. Connection to culture included prayer, dance, and song, all of which are traditional Lakota practices both in daily life and in ceremonies (Marshall, 2002). Although not specific to ACEs, research suggests that connection to Lakota culture, which included engaging in traditional practices, was related to lower rates of sexual harassment and physical dating violence victimization among middle and high school Native American girls (Edwards et al. 2020). Connection to culture has been linked to lower rates of caregiver substance use and caregiver mental illness (Kahn et al., 2016; Teufel‐Shone et al., 2018; Weaver, 2019; Yuan et al., 2015), which are ACEs. Thus, programs that seek to reconnect Native American families to culture may serve as an important tool in helping to prevent some ACEs among Native American children.

In addition to the connection to culture as a source of family strengths, participants identified engaging in healthy activities as a family way to help families be strong. Often this included activities outside, and the importance of eating meals with one another was also seen as important. This coincides with research suggesting that positive family behaviors, such as support, bonding, and closeness are protective against ACEs (Centers for Disease Control and Prevention Prevention., 2019; Stith et al., 2009). What is more, children with higher social‐emotional skills (e.g., impulse control, emotion regulation) may be at lower risk for experiencing some forms of ACEs, such as child physical and emotional abuse (Centers for Disease Control and Prevention Prevention., 2019). Although perpetrators are solely responsible for all acts of child abuse, research suggests that children with mental health issues are at increased vulnerability to be targeted for child abuse (Basile et al., 2016; Biglan et al., 2017). Thus, equipping youth with social‐emotional skills may reduce the likelihood that potential perpetrators will perceive them as vulnerable targets. In all, programs that seek to enhance family engagement in healthy activities may help to prevent some forms of ACEs among Native Americans.

Additionally, participants (especially caregivers) discussed the importance of keeping children safe as a requirement for families to be strong. This included strategies such as parental monitoring, which research suggests helps prevent against some forms of ACEs (e.g., child sexual abuse) as well as adolescent substance use and dating violence (Hebert et al., 2019; Rudolph & Zimmer‐Gembeck, 2018; Rudolph, Zimmer‐Gembeck, Shanley, & Hawkins, 2018; Rudolph, Zimmer‐Gembeck, Shanley, Walsh, et al., 2018). Thus, programs that teach caregivers parental monitoring skills may be effective in reducing some forms of ACEs. Additionally, caregivers noted the importance of teaching their children protective behavioral strategies, including self‐defense. Interestingly, research suggests that empowerment self‐defense training is effective in reducing sexual assault and sexual harassment victimization among Native American middle and high school girls (Edwards et al. 2020).

### 3.1 Limitations

Despite the important information learned from this study, several limitations should be noted. First, the sample size and we had challenges with participant engagement. However, the purpose of photovoice is not meant to have a large sample size for the purposes of generalizability but rather to prioritize “depth and breadth of information” for the purpose of “thematic data saturation” (Morton et al., 2020) which we believe was achieved in the current study. It is also important to note that many of the themes that emerged regarding family strengths have been stated as possible strategies to prevent ACEs (Centers for Disease Control and Prevention Prevention., 2019; Edwards et al., 2021). The themes that emerged in the current photovoice project align with other quantitative research finding the connection to Lakota culture and Lakota cultural identity as a protective factor for youth and family resilience (Edwards et al., 2021; Freeman et al., 2016) Another limitation is that we also had only mothers and grandmothers participate, although we welcomed other caregivers. Future research would benefit from engaging Native fathers and grandfathers in this type of work as well as caregivers who are Two‐Spirit or other gender diverse/trans identities. Finally, as previously noted, children did not engage to the extent that caregivers did in sessions until later sessions and thus the results may reflect the perspectives of caregivers more so of children although children all captured photographs representative of all of the themes presented for family strengths.

### 3.2 Program development

The findings from this photovoice project were used in conjunction with other data sources (e.g., focus groups with children, caregivers, practitioners) and extensive community input via an advisory board and informal conversations to create the Tiwahe Wicagwicayapi program. The Tiwahe Wicagwicayapi program is based off the Strengthening Families program, a strengths‐focused, family‐based program for 10‐ to 14‐year‐olds and their caregivers to prevent substance use initiation. The community felt that the Strengthening Families program, which has been adapted for Native American families (e.g., Bii‐Zin‐Da‐De‐Dah), was a good foundation from which to start given the program's focus on family strengths and the importance of family in Lakota culture.

The Tiwahe Wicagwicayapi includes seven group‐delivered sessions, each of which is named after a Lakota virtue. The program is for children ages 10–14 and their caregivers (broadly defined). The first four sessions, heavily based on the Strengthening Families program, focus on building family bonding and cohesion as well as communication and emotion regulation skills. Sessions five and six focus specifically on preventing child abuse (e.g., self‐protective strategies for children, parental monitoring strategies for caregivers) and draw from technical assistance packages from the CDC (Basile et al., 2016; Centers for Disease Control and Prevention Prevention., 2019) and other sources (Edwards et al., 2021). The final session (*wayunonihan*, Honor) focuses on honoring the hard work that the families have done over the past 7 weeks and includes a graduation celebration. All sessions integrate cultural components (e.g., talking circles, prayer, smudging, Lakota language) and skills‐based components. Further, skills‐based components are intentionally linked to Lakota cultural virtues. For example, children are told that shouting “No” and running away to unwanted sexual advances are consistent with the Lakota virtues of *waohola* (respect) and *woohitike* (bravery).

### 3.3 Concluding thoughts

In sum, the current study provides important insights into family strengths as identified by Native American children and their caregivers which include connection to culture, spending time doing healthy activities with one another, and keeping children safe, all of which were used to inform the development of a culturally grounded, strengths‐focused, family‐based program (i.e., Tiwahe Wicagwicayapi) to prevent ACEs. The current study also identified lessons learned from the first‐ever implementation of intergenerational photovoice with Native Americans. Finally, these findings further highlight that despite tremendous adversities, Native Americans are highly resilient and that family and connection to culture are critical sources of this resilience. As one participant stated: “Always family. You're still family, no matter what.”

## CONFLICTS OF INTEREST

The authors declare no conflicts of interest.
